# Correction: Association between breastfeeding, host genetic factors, and calicivirus gastroenteritis in a Nicaraguan birth cohort

**DOI:** 10.1371/journal.pone.0304878

**Published:** 2024-05-31

**Authors:** Nadja Alexandra Vielot, Ruthly François, Emilya Huseynova, Fredman González, Yaoska Reyes, Lester Gutierrez, Johan Nordgren, Christian Toval-Ruiz, Samuel Vilchez, Jan Vinjé, Sylvia Becker-Dreps, Filemon Bucardo

In Table 3, in the fifth column (Sapovirus, Adjusted) and the thirteenth row (Secretor+ vs. Secretor-) the value is incorrect. The correct value is 0.46 (0.17, 1.27). Please see the correct Table 3 here.

**Table 3:** Crude and adjusted relative hazards between breastfeeding and norovirus and sapovirus AGE in first 12 months of life.

	Norovirus AGE (n = 69)		Sapovirus AGE (n = 34)	
	Crude HR	Adjusted HR^1^	Crude HR	Adjusted HR^1^
Breastfeeding in the last week				
Any vs. none	1.31 (0.78, 2.21)	1.09 (0.62, 1.92)	0.99 (0.84, 1.17)	1.00 (0.82, 1.21)
Household sanitation				
Toilet vs. latrine or no sanitation	0.56 (0.35, 0.91)	0.66 (0.38, 1.12)	0.58 (0.29, 1.16)	0.46 (0.21, 1.02)
Consumption of high-risk foods in the last month				
Any vs. none	0.53 (0.29, 0.98)	0.69 (0.34, 1.41)	0.35 (0.16, 0.78)	0.36 (0.14, 0.90)
Child’s HBGA profile				
Secretor+ vs. Secretor-	9.60 (1.33, 69.15)	11.02 (1.50, 80.80)	0.92 (0.32, 2.60)	1.71 (0.39, 7.51)
Lewis+ vs Lewis-	0.97 (0.49, 1.89)	0.88 (0.40, 1.94)	1.28 (0.45, 3.62)	1.63 (0.36, 7.27)
Mother’s HBGA profile				
Secretor+ vs. Secretor-	0.81 (0.45, 1.44)	0.55 (0.29, 1.05)	0.68 (0.28, 1.62)	0.46 (0.17, 1.27)
Lewis+ vs Lewis-	1.11 (0.63, 1.97)	1.27 (0.66, 2.46)	1.22 (0.49, 3.06)	1.60 (0.55, 4.68)
HR = Hazard ratio				
^1^Hazard ratios adjusted for all other variables in the table.				
