# Supplementary material for: Association between breastfeeding, host genetic factors, and calicivirus gastroenteritis in a Nicaraguan birth cohort
Source: PLoS One. 2022 Oct 14;17(10):e0267689. doi: 10.1371/journal.pone.0267689 (PMC9565698; doi:10.1371/journal.pone.0267689)
Supplement: Attachment — Submitted filename: response to reviewers.docx [file pone.0267689.s004.docx]

**Journal Requirements**

1. Please ensure that your manuscript meets PLOS ONE's style requirements, including those for file naming. The PLOS ONE style templates can be found at

https://journals.plos.org/plosone/s/file?id=wjVg/PLOSOne_formatting_sample_main_body.pdf and

<https://journals.plos.org/plosone/s/file?id=ba62/PLOSOne_formatting_sample_title_authors_affiliations.pdf>

*The manuscript format has been updated according to the suggested style templates.*

2. Thank you for stating in your Funding Statement:

"This study was supported by award R01AI127845 from the National Institute of Allergy and Infectious Diseases (NIAID). SBD is supported by K24AI141744 from NIAID; FG, YR, and LG are supported by an international research capacity-building award from the NIH-Fogarty International Center, D43TW010923. "

Please provide an amended statement that declares *all* the funding or sources of support (whether external or internal to your organization) received during this study, as detailed online in our guide for authors at http://journals.plos.org/plosone/s/submit-now. Please also include the statement “There was no additional external funding received for this study.” in your updated Funding Statement.

Please include your amended Funding Statement within your cover letter. We will change the online submission form on your behalf.

*All funding has been declared, and the statement was amended above to clarify no additional funding sources.*

3. In your Data Availability statement, you have not specified where the minimal data set underlying the results described in your manuscript can be found. PLOS defines a study's minimal data set as the underlying data used to reach the conclusions drawn in the manuscript and any additional data required to replicate the reported study findings in their entirety. All PLOS journals require that the minimal data set be made fully available. For more information about our data policy, please see http://journals.plos.org/plosone/s/data-availability.

Upon re-submitting your revised manuscript, please upload your study’s minimal underlying data set as either Supporting Information files or to a stable, public repository and include the relevant URLs, DOIs, or accession numbers within your revised cover letter. For a list of acceptable repositories, please see http://journals.plos.org/plosone/s/data-availability#loc-recommended-repositories. Any potentially identifying patient information must be fully anonymized.

Important: If there are ethical or legal restrictions to sharing your data publicly, please explain these restrictions in detail. Please see our guidelines for more information on what we consider unacceptable restrictions to publicly sharing data: http://journals.plos.org/plosone/s/data-availability#loc-unacceptable-data-access-restrictions. Note that it is not acceptable for the authors to be the sole named individuals responsible for ensuring data access.

We will update your Data Availability statement to reflect the information you provide in your cover letter.

*Minimum analytic datasets for the norovirus and sapovirus analyses have been uploaded with this resubmission, as well as a codebook. Please update the data availability statement as suggested.*

4. We note that you have stated that you will provide repository information for your data at acceptance. Should your manuscript be accepted for publication, we will hold it until you provide the relevant accession numbers or DOIs necessary to access your data. If you wish to make changes to your Data Availability statement, please describe these changes in your cover letter and we will update your Data Availability statement to reflect the information you provide.

*See #4 above.*

5. Please review your reference list to ensure that it is complete and correct. If you have cited papers that have been retracted, please include the rationale for doing so in the manuscript text, or remove these references and replace them with relevant current references. Any changes to the reference list should be mentioned in the rebuttal letter that accompanies your revised manuscript. If you need to cite a retracted article, indicate the article’s retracted status in the References list and also include a citation and full reference for the retraction notice.

*All references have been reviewed and updated to follow Vancouver citation format, and author details for the following reference have been corrected:*

*4. Grant LR, O’Brien KL, Weatherholtz RC, Reid R, Goklish N, Santosham M, et al. Norovirus and Sapovirus Epidemiology and Strain Characteristics among Navajo and Apache Infants. PLoS One. 2017;12(1):e0169491.*

**Reviewer #1**

This study provides important information on associations between breastfeeding and acute gastroenteritis (AGE) caused by norovirus and sapovirus among children in a birth cohort in Leon, Nicaragua. This study has very rich weekly data on breastfeeding and AGE and monthly data on other risk factors, enabling the authors to conduct a time-varying Cox hazard analysis. Results are interesting. Some of them align with previous findings and others don’t, and the authors provide good discussion on each point, which was helpful. I have a few comments.

Results
- Regarding the protective effect of the consumption of high-risk foods and eating food outside, I agree that it is surprising. The authors discuss potential reasons behind this in the Discussion, which was helpful. Could you also include an interaction term between the variable for the comsumption of high-risk foods and the variable for the socioeconomic status in your Cox hazard model? That might be helpful in interpreting the relationship.

*We agree with this suggestion, and had performed this analysis initially. We found a non-significant interaction between having a toilet in the home and consumption of high-risk food, which is the proxy economic variable that we chose for our model. We have added this finding to the results to clarify:*

*Lines 199-200: “For both pathogens, interaction terms between nutrition and socioeconomic factors were not statistically significant.”*

*We did, however, find associations between consumption of high-risk foods and presence of piped water in the house, which we had previously noted in the discussion. This finding suggests that consumption of certain foods might indeed be associated with economic factors, but that not all economic factors measure the same constructs or serve as indicators for nutritional patterns.*

Discussion
- I do not know any background in Nicaragua, and did not understand why exclusive breastfeeding was associated with travel time to a health center. What does a health center/post do with breastfeeding?

*We have clarified the association with distance to a health center as a proxy for rural residence as follows:*

*Lines 230-236: “A prior study of breastfeeding practices in León found only 12.7% exclusive breastfeeding for six months, and indicated that travel time to a health center greater than one hour, as a proxy measure for living in a remote or rural area, was associated with exclusive breastfeeding in the first six months of life [29]. In León, most women have access to a health post in their neighborhoods, suggesting that exclusive breastfeeding is less common in urban-dwelling women, and might be associated with greater access to infant formulas and other feeding options.”*

- Are there any studies or articles you could cite about cultural and social factors associated with short-term exclusive breastfeeding that you talked about in LIne 236-238 (e.g., prolonged breastfeeding negatively affecting breast shape and volume)?

*We have added two citations referring to cultural and social norms around breastfeeding in Latin American women in lines 244-246.*

- I guess a natural question from readers would be “so what about other pathogens tested in this study?” but I guess the authors have a plan to write another report on that.

*This is correct, and we have clarified that we have plans for future studies to assess the role of co-infections in AGE and potential associations with breastfeeding:*

*Lines 288-291: “It is possible that breastfeeding is associated with protection against other enteric pathogens that we did not assess in the current analysis. Ongoing studies are assessing pathogen-specific associations with breastfeeding across a wider array of pathogens, accounting for the composition of breastmilk with respect to IgA antibodies and HMOs as potential protective factors against AGE.”*

**Reviewer #2:**

In this study, Vielot and coauthors analyzed the association between breastfeeding practices, child/mother genetic factors, and norovirus/sapovirus AGE using a birth cohort data obtained in Nicaragua. Authors found strong associations between child secretor status and norovirus infections in their first year of life but authors could not find conclusive evidence of associations between breastfeeding and protection against AGE caused by norovirus and sapovirus due to a limited sample size and the fact that majority of mothers performed exclusive breastfeeding for only a few weeks.

While this study could not provide conclusive evidence of associations between breastfeeding and viral AGE, findings provided in this study are important to further conduct and understand the roles of exclusive and non-exclusive breastfeeding on norovirus/sapovirus AGE. Thus, it implies that non-exclusive breastfeeding may not protect children from norovirus/sapovirus AGE, and warrants further studies with different population where exclusive breastfeeding is performed commonly for a long duration or under the lactation support. Please find below the comments/suggestions to improve the manuscript before publication.

1. Authors claimed that "most norovirus episodes occurred among secretor positive and Lewis positive children...", but it could be confusing and mislead an incorrect conclusion as most of the children enrolled and studied in this cohort were secretor positive and Lewis positive. In fact, authors analyzed the HR with host genetic factors in Table 3 to show that there is no association between Lewis profiles and norovirus/sapovirus AGE. I would suggest to rephrase the section "Maternal and child HBGA profiles" or omit or combine this section with the next section.

*Thank you for this suggestion. We have modified Table 2 to show additional data to help the reader contextualize the distribution of phenotypes and associations with disease. The table now includes bivariate comparisons of each secretor and Lewis status with each norovirus and sapovirus infection status, for children and their mothers. We have also added text to the results as follows:*

*Lines 183-186. “For norovirus, we observed inverse associations between secretor-positive and secretor-negative children in risk of norovirus AGE; this association was less pronounced with respect to maternal secretor status, and no such associations were observed for sapovirus (Table 2).”*

2. One of the interesting findings in this study is that they did not find any associations between host genetic factors (neither secretor nor Lewis) and sapovirus AGE. Is there any other studies that indicate the roles (or no roles) of host genetic factors on sapovirus infections? If so please consider to include findings from such previous studies and provide more discussions on the roles of secretor status on sapovirus infections.

*Thank you for this suggestion. We have cited prior studies, including from this research group, showing consistent findings of no association of HGBA profiles with risk for sapovirus, and updated the discussion as follows:*

*Lines 218-222: “These influences of secretor status were not observed for sapovirus AGE, consistent with findings from prior studies indicating that sapovirus infects individuals of all HBGA phenotypes [18,24,25]. As sapovirus does not bind to HGBAs, there is presumably no influence of HBGA status on sapovirus infection risk [26].”*

3. Another interesting observation is that consumption of high-risk foods was protective against sapovirus AGE. Authors suggested that it could be due to confounding factors such as nutritional practices or socioeconomic status, and I agree with authors but please also consider the epidemiological observations that foodborne ourbreaks are less common in sapovirus as compared to norovirus. I believe there is no conclusive evidence on the risk of food consumption on sapovirus AGE, but if the risk is lower in sapovirus than in norovirus, it may explain the differences of the observation between norovirus AGE and sapovirus AGE in this study. Thus, it showed significant protective associations only among sapovirus AGE but not in norovirus AGE as food consumption could work as a risk factor only (or more) in norovirus AGE.

*Thank you for this helpful explanation, which we have incorporated into the discussion with relevant citations as follows:*

*Lines 254-257: “Sapovirus infections are usually transmitted by the fecal-oral route, and are less likely to be caused by foodborne outbreaks compared to norovirus [34]. This might explain why the risk of sapovirus AGE was not affected by adjusting for consumption of high-risk foods, whereas protective association with norovirus was attenuated and nullified on adjustment.”*

4. I understand typing of host genetic factors was performed in their previous study, but please consider to describe again in this manuscript how the host genetic factors were determined in the method section.

*We have described the phenotyping process in the methods section as follows:*

*Lines 128-135: “Secretor and Lewis phenotypes were determined using an in-house saliva-based ELISA as described previously [21]. In brief, secretor antigens present in saliva were recognized using the Ulex europaeus Lectin peroxidase (UEA-I, Sigma-Aldrich, Sweden) and Lewis antigens using monoclonal anti-Lewis-a and anti-Lewis-b from Seraclone and Diaclone (Bio-Rad, Uppsala, Sweden), respectively. Children with optical reading for Lewis a or Lewis b were classified as Lewis positive (Le +). If the result was indeterminate, we tested the samples collected at 12 or 24 months of age. Saliva samples from mothers were collected at the end of the study and tested for HBGAs phenotypes [22].”*

**Reviewer #3:**

Violet et al studied the association between calicivirus AGE and exclusive breastfeeding or host HBGA status in a Nicaragua birth cohort from June 2017 to July 2018. This study was very well planned out and its findings on how maternal and child secretor status influences norovirus disease risk is not unexpected, nevertheless very interesting. This manuscript is very well written.

Minor questions and comments:
Were there seasonal trends in sapovirus and norovirus positivities? Were they consistent with what's typically seen in the region?

*Seasonality is now described in the results, though data only span 1 full year and cannot show clear trends over time. We have included an additional figure to illustrate the timing of norovirus and sapovirus infections.*

*Lines 161-163: “The first peak of norovirus infections occurred in January 2018, with subsequent peaks in mid- and late-2018 (Fig 2). A single peak was identified for sapovirus, in mid-2018.”*

Line 181-182, diarrhea lasting more than 7 days is not considered as acute gastroenteritis in most clinical definitions. Consider reword/or update definition of AGE in methods.

*Thank you for this clarification. We have clarified that persistent gastroenteritis occurred in a small number of episodes that persisted for more than 14 days.*

*Lines 174-176: “Four of the 69 norovirus episodes (5.8%) and three of the 34 sapovirus episodes were associated with diarrhea lasting more than 14 days, thereby becoming persistent gastroenteritis [23].”*

Line 201-202, If secretor positive were a dominate trait, it would means 3/4 progeny would have the trait from AaXAa heterozygous parents, all progeny if one parent is AA and 1/2 if from AaXaa parents. I wouldn't call it surprising to see more secretor positive children than mothers. Also, it is a bit more complicated than a simple Mendelian hereditary pattern since there are weak secretor alleles that are semi-functional. I would suggest not to get into the genetics of HBGA here.

*Thank you for providing this helpful explanation. We have removed the reference to Mendelian heredity.*
